# Diffusion Tensor Histogram Analysis of Pediatric Diffuse Intrinsic Pontine Glioma

**DOI:** 10.1155/2014/647356

**Published:** 2014-06-11

**Authors:** Emilie A. Steffen-Smith, Joelle E. Sarlls, Carlo Pierpaoli, Joanna H. Shih, Robyn S. Bent, Lindsay Walker, Katherine E. Warren

**Affiliations:** ^1^Pediatric Oncology Branch, National Cancer Institute, Center for Cancer Research, National Institutes of Health, Building 10, Room 1-5750, 9000 Rockville Pike, Bethesda, MD 20892, USA; ^2^In Vivo NMR Center, National Institute of Neurological Disorders and Stroke, National Institutes of the Health, Bethesda, MD 20892, USA; ^3^Program on Pediatric Imaging and Tissue Sciences, National Institute of Child Health and Human Development, National Institutes of Health, Bethesda, MD 20892, USA; ^4^Biostatistics and Data Management Section, National Cancer Institute, Center for Cancer Research, National Institutes of the Health, Bethesda, MD 20892, USA; ^5^Center for Biomedical Engineering, School of Engineering, Brown University, Providence, RI 02912, USA

## Abstract

*Purpose*. To evaluate tumor structure in children with diffuse intrinsic pontine glioma (DIPG) using histogram analyses of mean diffusivity (MD), determine potential treatment and corticosteroid-related effects on MD, and monitor changes in MD distributions over time. *Materials and Methods*. DTI was performed on a 1.5T GE scanner. Regions of interest included the entire FLAIR-defined tumor. MD data were used to calculate histograms. Patterns in MD distributions were evaluated and fitted using a two-normal mixture model. Treatment-related effects were evaluated using the *R* ^2^ statistic for linear mixed models and Cox proportional hazards models. *Results*. 12 patients with DIPG underwent one or more DTI exams. MD histogram distributions varied among patients. Over time, histogram peaks became shorter and broader (*P* = 0.0443). Two-normal mixture fitting revealed large lower curve proportions that were not associated with treatment response or outcome. Corticosteroid use affected MD histograms and was strongly associated with larger, sharper peaks (*R* ^2^ = 0.51, *P* = 0.0028). *Conclusions*. MD histograms of pediatric DIPG show significant interpatient and intratumoral differences and quantifiable changes in tumor structure over time. Corticosteroids greatly affected MD and must be considered a confounding factor when interpreting MD results in the context of treatment response.

## 1. Introduction

Pediatric diffuse intrinsic pontine gliomas (DIPGs) are highly invasive, aggressive lesions that infiltrate the pons. The location and infiltrative nature of DIPG precludes surgical intervention. Diagnostic biopsy is controversial and not routinely performed at most institutions for patients with a typical presentation, resulting in a paucity of histological data and limited understanding of DIPG biology at diagnosis [1, 2]. Studies at autopsy indicate that the majority of DIPGs are high-grade with substantial interpatient variation in gene expression and molecular genetic aberrations [3–5]. Imaging remains the primary modality for diagnosis, assessment of therapeutic response, and management. However, standard MRI findings, including enhancement and tumor size measurements, are difficult to interpret, obtain consistently, and provide little insight into underlying tumor structure and biology. Changes on standard MRI are not specific to response or outcome [6–11]. Given the limited utility of standard MRI and the heterogeneous nature of DIPG, advanced imaging techniques, including diffusion tensor imaging (DTI), are currently under investigation to interrogate the structure and behavior of DIPG [12, 13]. Most studies using DTI to evaluate brain tumors have used measurements of the average value of mean diffusion (MD) from a specific region to represent the entire tumor. MD is more sensitive to changes in cellularity and edema compared to fractional anisotropy (FA), which may increase or decrease depending upon the FA of the original structure [14]. Such regional analyses rely partly on* a priori* knowledge of boundaries between tissue subtypes (e.g., active tumor and necrosis), which may have different MD values [15]. DIPGs have indistinct borders and may contain areas of varying tumor activity, normal grey and/or white matter, edema, and necrosis, making it difficult to identify a region that best characterizes the tumor. In addition, DTI in the brainstem is especially challenging due to geometric distortions found at the air-tissue interface of the paranasal sinuses (i.e., susceptibility artifacts) and movement from cardiac pulsation (i.e., motion artifacts), both of which may produce spurious diffusion results [16]. A histogram can provide analysis of MD values across the entire tumor volume, giving a graphic, quantitative representation of the distribution of MD values from intratumoral heterogeneity. Changes in the distribution of MD values in different tumor regions may reflect changes in tissue subtypes over the course of treatment (e.g., increase in proportion of necrotic tissue compared to proportion of active tumor). MD histogram analyses have been used to differentiate high-grade and low-grade tumors in adults [17, 18]. Studies of adult high-grade gliomas have used MD histograms to predict patient outcome and treatment response [19–22]. Patients in these studies frequently receive corticosteroids, with variable doses among patients [19, 21, 22]. Corticosteroids have a known effect on diffusion parameters, reducing the magnitude of diffusion within brain tumors [23, 24]; yet reports of MD histograms in adults with gliomas have not accounted for corticosteroid use when interpreting treatment-response. Corticosteroids are commonly used to manage clinical symptoms in children with DIPG. This study used a comprehensive DTI acquisition and processing method to determine if global MD measures and MD histograms could aid in assessment of treatment effects by determining corticosteroid-related changes and longitudinal changes in DIPG.

## 2. Methods

### 2.1. Patients

Patients or their legal guardians signed a document of informed consent for enrollment in a phase II study of Pegylated Interferon Alfa-2b (PEG-Intron) for children with DIPG [25]. The institution review board approved the study. Study eligibility criteria are described in Warren et al.'s [25]. Patients were required to enroll within 2–10 weeks after completion of radiation treatment and, if receiving corticosteroids, maintain a steady or decreasing dose for ≥1 week prior to study entry. DTI was acquired during standard MRI evaluations on a subset of patients.

### 2.2. MRI

Imaging data were acquired on a single GE Signa HDx 1.5T scanner (GE Medical Systems, Milwaukee WI) equipped with an eight-channel phased array coil. Clinical imaging sequences included pre- and postcontrast T1 spin echo (TR/TE = 450/13 ms, FOV = 220 × 220 mm, matrix = 256 × 192, thickness = 3 mm), T2-fast spin echo (T2-TSE; TR/TE = 3400/95 ms, FOV = 220 × 220 mm, matrix = 256 × 192, slice thickness = 3 mm), and fluid attenuated inversion recovery (FLAIR; TR/TE/TI = 10,000/140/2200 ms, FOV = 220 × 220 mm, matrix = 256 × 192, thickness = 3 mm). Precontrast whole-brain DTI datasets were acquired using a dual spin-echo preparation period and single shot spin-echo echo planar imaging (EPI) sequence (TR/TE = 17.6/89.3 ms, FOV = 240 × 240 mm, matrix = 96 × 96, thickness = 2.5 mm, no gap, 64 slices). Diffusion gradient encoding was applied in 60 noncollinear directions with maximum *b*-value = 1100 s/mm^2^ and in 10 noncollinear directions with *b*-value = 300 s/mm^2^ and *b*-value = 0 s/mm^2^ (80 imaging volumes total).

### 2.3. DTI Processing and Analysis

Diffusion data were processed offline using TORTOISE [26]. T2-FSE images were used as the structural target for DTI data processing. T2-FSE images from the first time point were aligned to the hemispheric midline and the anterior and posterior commissures plane using MIPAV [27]. Follow-up scans were registered to the first time point. Diffusion weighted-imaging (DWI) data were corrected for rigid body motion, eddy-current distortion [28], and EPI distortion [29]. Corrected DWI data were registered to T2-FSE structural images. The DT [30–32] was calculated using a nonlinear least squares method with robust estimation of tensors by outlier rejection [33], which removes physiological effects like cardiac pulsation. MD maps were calculated from the DT.

### 2.4. Regions of Interest (ROI) Analysis

**Figure 1:**
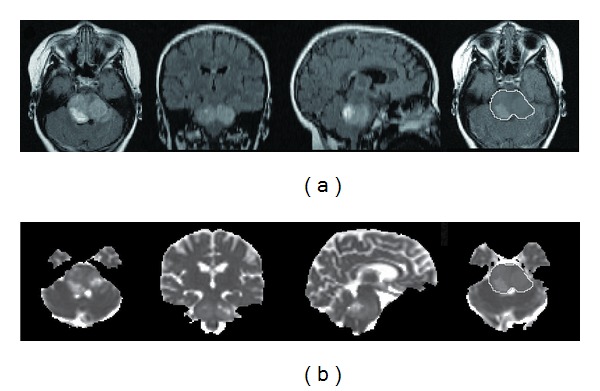
Coregistered axial, coronal, and sagittal (a) FLAIR images and (b) MD maps of the pons. ROIs were outlined on axial images ((a) and (b) far right) and combined to create a volumetric ROI covering the entire lesion.

Precontrast FLAIR images and MD maps were coregistered via the T2-TSE structural target using MIPAV and imported into TORTOISE for ROI analysis. FLAIR images were used for ROIs based on a previous report of more consistent selection of tumor boundaries with FLAIR compared to T2 [34]. Enhancement was not considered in ROI selection given the highly variable and frequently absent contrast enhancement patterns found in DIPG. Regions of FLAIR abnormality on each axial slice were used to determine tumor involvement within the pons and surrounding tissue. FLAIR signal abnormality was frequently diffuse, without distinct borders. Therefore, slice ROIs were manually drawn to include the entire FLAIR signal abnormality and the affected anatomical structure, excluding regions of CSF (Figure 1). ROIs were applied to MD maps. Axial ROIs were combined to create a volumetric ROI. Global MD measures from the volumetric ROI data included median, mean, 5th percentile (lowest 5% of ROI values), and 95th percentile (highest 5% of ROI values).

### 2.5. DTI Histograms

MD values from volumetric ROIs were used to generate MD histograms, plotting the frequency of MD values as a proportion of total ROI voxels versus MD values (bin width of 0.025 × 10^−3^ mm^2^/s). Analysis of MD histogram characteristics included (1) standard deviation; (2) skewness, measure of histogram asymmetry: length of left tail > right tail (negative skewness) or length of right tail > left tail (positive skewness), (3) peak location (mode), and (4) peak height. MD values were also fitted using a two-normal mixture distribution, a model reported in studies of adult glioblastoma [20–22]. From the two-normal mixture histograms we calculated the lower normal curve proportion (LCP), which represents the percentage of histogram data found within the lower curve, and the lower normal curve mean (LCM), that is, the mean MD value of the lower curve. Previous studies indicate that LCP reflects active, highly cellular areas of the tumor and the high normal curve proportion reflects necrotic or edematous regions [21, 22].

### 2.6. Statistical Analysis

We evaluated differences among patients using global MD measures and MD histogram characteristics at the first DTI scan and longitudinally. A linear mixed effect model was used with a random intercept and random slope to account for intraperson correlation due to multiple scans. We used an *R* ^2^ statistic for linear mixed models [35] to evaluate the association between MD measures and time. This same method was applied to evaluate the relationship between ROI volume and global and histogram parameters. Kaplan-Meier method was used to calculate the time to progression and overall survival relative to study entry. *R* ^2^ statistic for linear mixed models was used to examine the relationship between MD values and corticosteroid use, time to disease progression, and overall survival. We also applied univariate Cox proportional hazards models to explore the relationship of global measures and histogram parameters with progression and overall survival. *P* values of <0.05 were considered statistically significant. Data were analyzed using the statistical computing package, *R* (http://www.r-project.org/).

## 3. Results

### 3.1. Patients

Twelve patients (median age = 5 y, range = 4–8 y) underwent one or more DTI exams during the course of treatment with PEG-Intron (Table 1). Six patients received corticosteroids (dexamethasone), at the time of their initial DTI exam. Four of those patients continued to receive dexamethasone at subsequent time points, and, in each case, the dose was stable or decreased from the previous time point. Median time to disease progression was 28.1 weeks from study entry. Median overall survival was 45.7 weeks from study entry.

### 3.2. Global MD Measures

**Figure 2:**
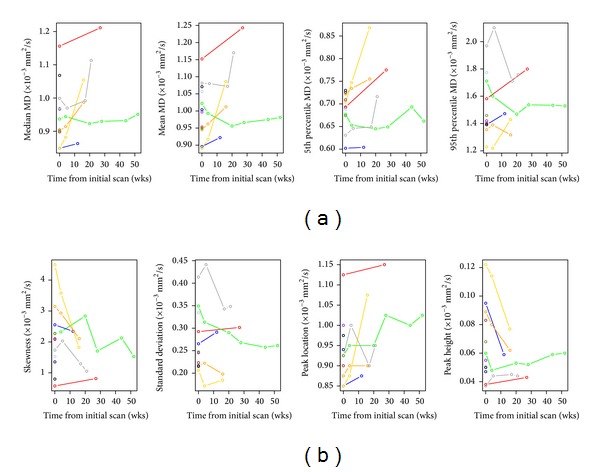
(a) Changes in global MD parameters over time for all patients. (b) Changes in MD histogram parameters over time for all patients.

Global MD measures for all time points are shown in Figure 2(a). Median and mean MD values from the initial DTI scan were increased compared to those of normal brain tissue (MD of normal tissue = 0.7 × 10^−3^ mm^2^/s), with considerable variability among patients: median MD range = 0.85–1.16 × 10^−3^ mm^2^/s and mean MD range = 0.9–1.17 × 10^−3^ mm^2^/s, respectively. Median and mean MD increased significantly over time (*R* ^2^ = 0.29, *P* = 0.0369 and *R* ^2^ = 0.28, *P* = 0.0427, resp.). The prognostic value of the median and mean MD did not reach statistical significance (*P* > 0.1). Fifth percentile MD values also increased significantly over time (*R* ^2^ = 0.35, *P* = 0.0202), with lower values at scans closer to study entry (*R* ^2^ = 0.31, *P* = 0.0316). Fifth percentile MD values appeared to be a strong predictor of progression and overall survival (HR > 20) but did not reach statistical significance (*P* > 0.1).

### 3.3. MD Histogram Measures

**Figure 3:**
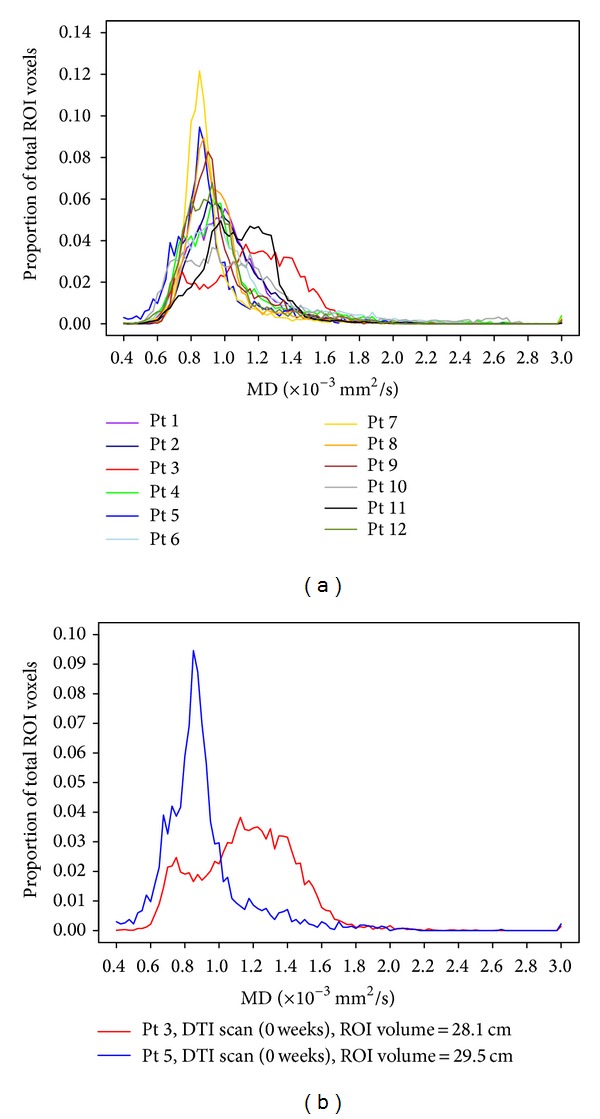
(a) Baseline MD histograms for all patients. (b) Baseline MD histograms for two patients, showing interpatient heterogeneity of baseline MD. DTI scans were performed two weeks after XRT and prior to PEG-Intron therapy for both patients. Both patients received dexamethasone at the time of scan.

**Figure 4:**
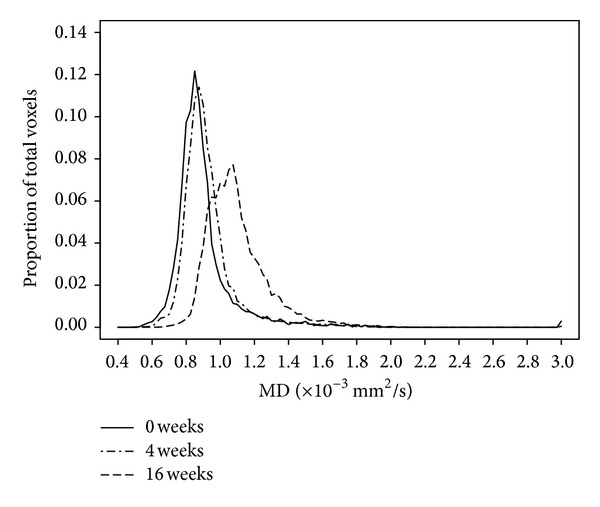
Changes in MD histogram over time for an individual patient.

MD histogram measures for all time points are shown in Figure 2(b). MD histograms from initial DTI scans revealed heterogeneous distributions of MD values within each lesion, with no consistent histogram shape among patients (Figure 3(a)). Large differences in histogram shape and distribution were observed, even among patients at the same stage of their clinical course (Figure 3(b)). At subsequent scans, we continued to observe interpatient variation in histogram shape and distribution. As illustrated in Figure 4, changes in histograms for individual patients over time included a shift towards higher MD values and decreased peak height (i.e., shorter, broader peaks). Peak height was negatively associated with time from initial scan (*R* ^2^ = 0.28, *P* = 0.0443) and appeared to be a potentially strong predictor of progression and overall survival (HR > 20), but the association did not reach statistical significance (*P* > 0.1). Histogram standard deviation was associated with time from initial scan (*R* ^2^ = 0.44, *P* = 0.0074) and was lower in patients with scans closer to study entry (*R* ^2^ = 0.44, *P* = 0.007).

**Figure 5:**
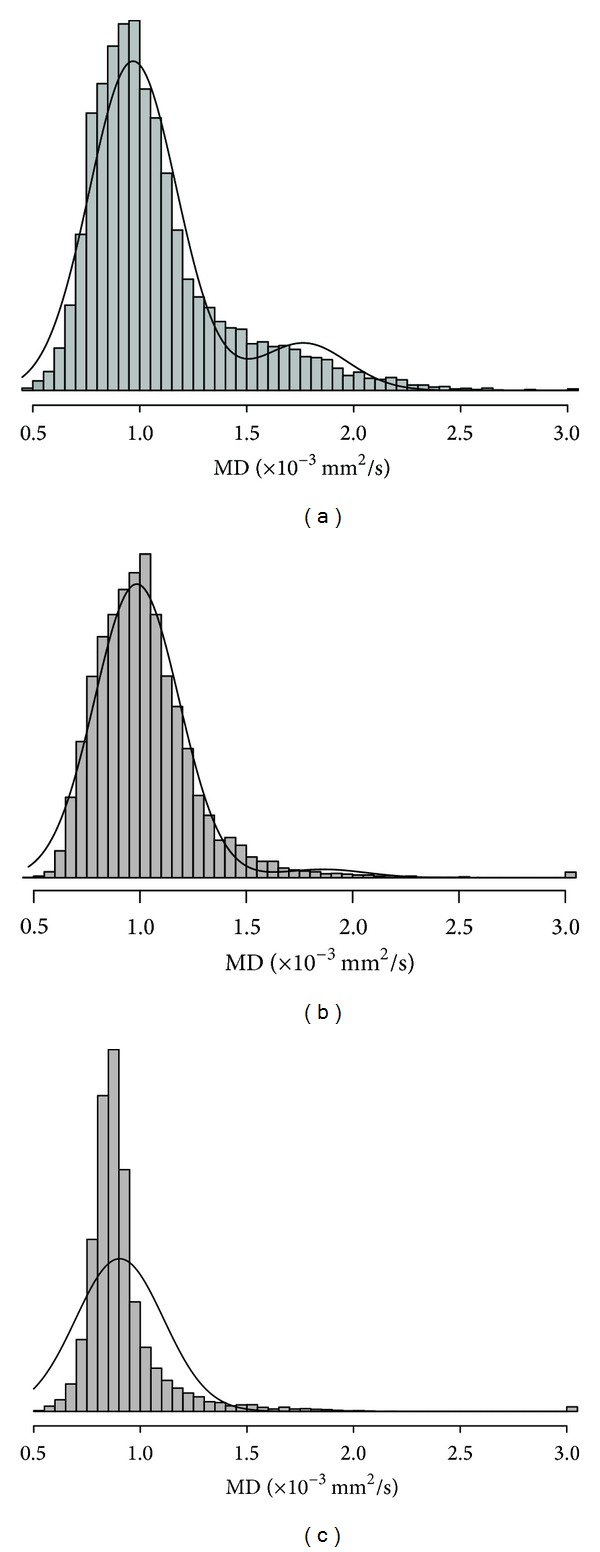
Two-normal mixture fitting for 3 patients on study. Overall, two-normal mixture histograms had large lower curves (a, b) with an acceptable overall fit to the distribution of data. However, for one patient with a very narrow peak (c), the model was not appropriate and excluded a large proportion of MD values.

Unlike reports in adults, the two-normal mixture fitting of MD data resulted in large lower curves which included an overwhelming majority of MD histogram values for nearly all patients (median LCP = 92.98%, SD = 17.11%), with the exception of one patient in which the model was a poor fit for the histogram data (Figure 5). LCM MD values were higher than normal tissue for all patients, ranging from 0.88 to 1.16 × 10^−3^ mm^2^/s (median = 0.95 × 10^−3^ mm^2^/s, SD = 0.09 × 10^−3^ mm^2^/s). Prognostic values of LCP and LCM at initial scan (HR < 1) and over time (HR < 1 and HR < 5, resp.) were not statistically significant (*P* > 0.1 for all analyses).

### 3.4. ROI Volume Measure

Volumetric ROIs ranged from 9.0 cm^3^ to 63.6 cm^3^. For patients with follow-up DTI, ROI volumes typically increased over time, though this trend did not reach statistical significance (*P* > 0.05). Larger ROI volume was positively associated with increased time from study entry (*R* ^2^ = 0.28, *P* = 0.0443). ROI volume was inversely associated with positive histogram skewness (*R* ^2^ = 0.27, *P* = 0.0478) and positively associated with median MD (*R* ^2^ = 0.52, *P* = 0.0025) and mean MD (*R* ^2^ = 0.51, *P* = 0.0027). We identified a strong positive linear relationship between fifth percentile MD and ROI volume (*R* ^2^ = 0.68, *P* < 0.001), suggesting that larger tumors are more edematous or necrotic compared to smaller tumors. Clinical parameters, including use of corticosteroids, were not associated with ROI volume (*P* > 0.05).

### 3.5. Use of Corticosteroids and MD

Several differences in MD values were found for those patients receiving corticosteroids compared to those who were not (Table 2). Dexamethasone use was associated with lower median MD (*R* ^2^ = 0.43, *P* = 0.0182) and lower mean MD (*R* ^2^ = 0.38, *P* = 0.0077). MD histograms for patients receiving dexamethasone showed significantly more positive skewness (*R* ^2^ = 0.38, *P* = 0.0136) and increased peak heights (*R* ^2^ = 0.51, *P* = 0.0028) compared to those of patients not receiving dexamethasone.

## 4. Discussion

With the increasing push for biopsy and development of targeted therapies for DIPG, more insight into the tumor environment is critical. In this study, we show that MD histogram analysis allowed further diffusion-related changes to be quantified and monitored over time compared to global ROI measures. Even in this small number of patients, we observed heterogeneous distributions of MD values, likely reflecting the known substantial interpatient biologic variation in DIPGs [3–5] and showed dynamic changes in tumor structure over time. Shape and appearance of MD histograms among patients varied greatly, even among patients at the same point in their treatment course. Histogram shape changed over time, though the observed patterns of change differed among patients. We observed a shift in histograms towards larger MD values and a decrease in peak height over time, particularly for patients with larger peak heights at their first DTI scan. This suggests an increase in tumor heterogeneity following treatment. We consistently saw an increase in MD values, though the relationship between MD values and progression and survival was not statistically significant. Increased MD was seen even in fifth percentile MD values, which represent regions with the lowest MD and, presumably, areas of greatest cellularity within the tumor ROI. The clinical significance of these findings is unknown but demonstrates that the tumor environment is dynamic, changing over time and course of therapy. Larger MD values could reflect an increase in extracellular water content compared to normal tissue either due to interstitial edema or the formation of cystic cavities associated with necrosis.

Studies in adult glioblastoma have applied a two-normal mixture model to MD histogram data and demonstrated a better fit of MD data and improved analysis for histogram measures, using MD values from the lower curve to stratify patients and predict treatment response [20–22]. For comparison, we applied the same model to our pediatric DIPG MD histogram data. Unlike reports in adults, the two-normal mixture model did not improve our analysis of pediatric DIPG. We observed relatively similar, large lower curves for almost all patients at all-time points and found no significant association between lower curve metrics and outcome.

In this study, changes in MD were clearly associated with use of corticosteroids. The effect of corticosteroids on diffusion properties in treatment-naïve adult patients with high-grade gliomas has been previously evaluated [23, 24], showing a decrease in MD with administration of corticosteroids. However, the effect on diffusion characteristics in patients undergoing concurrent treatment was previously unreported. Consistent with results from previous studies, diffusion parameters were significantly different when patients received dexamethasone, even in those patients receiving a steady or tapering dose. The primary effect of corticosteroids is a reduction or resolution of edema in tissue, reflected by overall lower MD values. Results from our study indicate that corticosteroid use in patients receiving antitumor therapy greatly impacts the DTI results and must be considered a confounding factor when using DTI to determine treatment response in this population.

Results of this study must be interpreted with consideration of limitations. Patients were enrolled in a clinical trial following standard radiation therapy; therefore, all DTI scans were performed following radiation and we were unable to assess changes in MD parameters before and after radiation therapy. Analysis of MD histograms to determine treatment response was limited by the number of patients who had longitudinal scans. We observed that over time the proportion of tissue with normal MD decreased in children with DIPG. The timing of DTI scans over the course of treatment was variable among patients due to scheduling limitations. Therefore, variation in MD histogram appearance at the initial scan cannot be solely attributed to differences in the tumors among patients, but also due, in part, to differences in the timing of scans relative to treatment. As is common in this population, use of corticosteroids varied among patients. We observed significantly lower MD values when corticosteroids were administered, which may reflect a reduction in edema within the tumor or may be a combination of the effect of corticosteroids and treatment response. Because correlation of diffusion parameters with tissue histology is not possible (due to restrictions on biopsy in children with DIPG), the biological interpretation of our findings is limited.

## 5. Conclusions

This study investigated both global MD measures, which are typically reported, and MD histogram characteristics, after comprehensive data processing, in children receiving treatment for DIPG. The most striking observations were the interpatient variation and intratumoral heterogeneity seen in MD and the significant effect of corticosteroids on MD. Our study shows that MD histogram analysis can be used to visualize the known heterogeneity of DIPGs* in vivo* and to objectively quantify changes in tumor microstructure over the course of therapy that may not be captured using a global measure of MD values or findings on standard MRI. In addition, we caution that corticosteroid use in patients concurrently receiving antitumor therapy should be considered a confounding factor when analyzing DTI data.

## Figures and Tables

**Table 1:** Patient characteristics.

Pt	Age (yr)	M/F	XRT (wks)	TTP (wks)	OS (wks)	DTI (wks)	Dexamethasone dose (mg/kg)
1	4	M	9	24	39	24	None
2	6	M	8	40	48	40	None
3	5	F	2	37	51	0	0.12
						27	0.11
4^1^	6	F	6	72	151+	12	None
						16	None
						32	None
						40	None
						56	None
						64	None
5	5	F	5	25	38	4	0.09
						16	0.01
6	5	F	9	9	23	4	0.08
7	6	F	5	16	31	0	0.01
						4	0.01*
						16	None
8	8	F	2	24	32	0	0.02
						4	0.01
						16	None
9	6	F	5	21	22	8	None
10	5	F	7	31	51	7	None
						12	None
						24	None
						28	None
11	4	M	4	16	24	4	0.27
12	5	F	8	15	49	0	None

^1^Patient went off study due to disease progression but continues to be followed.

XRT: radiation therapy (time from end of XRT to study entry).

TTP: time to disease progression (from study entry).

OS: overall survival (from study entry).

DTI: diffusion tensor imaging (time of scan from study entry).

*administered every other day.

**Table 2:** Use of corticosteroids and MD parameters.

MD parameter	No corticosteroids	+Corticosteroids
	Median (SD)	Median (SD)
Median MD (×10^−3^ mm^2^/s)	0.96 (0.06)	0.90 (0.13)
Mean MD (×10^−3^ mm^2^/s)	1.0 (0.06)	0.95 (0.12)
Skewness	2.06 (0.46)	2.45 (1.32)
Peak Height	0.05 (0.01)	0.07 (0.03)

SD: standard deviation.
